# Prevalence of unrecognized myocardial scarring in patients with normal contractile function across four U.S. hospitals

**DOI:** 10.1186/1532-429X-18-S1-O131

**Published:** 2016-01-27

**Authors:** Han W Kim, Dina Labib, Dipan J Shah, Afshin Farzaneh-Far, John Heitner, John D Grizzard, Anna Lisa Crowley, Igor Klem, Faisal Nabi, Raymond Kim, Robert Judd

**Affiliations:** 1 Cardiology/Medicine, Duke University Medical Center/Duke Cardiovascular Magnetic Resonance Center, Durham, NC USA; 2 grid.63368.380000000404450041Cardiology/Medicine, Methodist DeBakey Heart and Vascular CenteCardiovascular Imaging Institute, The Methodist Hospital, Houston, TX USA; 3 grid.185648.60000000121750319Cardiology, University of Illinois, Chicago, IL USA; 4 grid.415436.10000000404437314Cardiology, New York Methodist Hospital, Brooklyn, NY USA; 5 grid.224260.00000000404588737Radiology, Virginia Commonwealth University, Richmond, VA USA

## Background

Prior studies have established that myocardial scarring increases mortality even without overt symptoms of irreversible injury, for example due to unrecognized myocardial infarction (UMI) in patients with coronary artery disease (CAD), or replacement scarring in non-ischemic cardiomyopathy (NICM). Previous studies have also established that scar formation may be missed by echocardiography and SPECT due to normal wall motion and/or limited spatial resolution. CMR has a high sensitivity for the detection of scar, but it is currently unknown how many patients have unrecognized scar because no large-scale studies have addressed this question.

## Methods

Data analysis was performed on a cloud-based system that is currently receiving de-identified searchable data from electronically-signed clinical reports with full DICOM datasets for 23,275 consecutive CMR exams performed at four U.S. hospitals from Jan 1, 2010 through Dec 31, 2014. At the time of abstract submission, 8,242 datasets have been analyzed, and analysis of all 23,275 is expected by the end of 2015. All data fields were derived from CMR reports that had been electronically signed by board-certified physicians with Level 3 CMR training. All patients with a history of MI were excluded. Normal contractile function was defined as normal wall motion scores in all 17 myocardial segments. Scar was defined as the presence of delayed enhancement in post-contrast images. Scar pattern (CAD or non-CAD) was defined by the physician's interpretation in the electronically signed final clinical report.

## Results

Of 8,242 consecutive patients undergoing clinical CMR for routine purposes (indications: cardiomyopathy 26%; ischemia evaluation 19%; vascular 18%; viability assessment 14%; arrhythmia 12%), 4,110 patients had normal wall motion scores and no history of MI. Patient demographics are shown in Table 1. Of these 4,110 patients, scar was present in 543 (13.2%). Ejection fraction was normal in patients with scar (64 ± 9%) and without scar (62 ± 7%). In the 543 patients with scar, 199 (37%) had a scar pattern consistent with UMI from CAD, while the remainder had non-CAD patterns of scar. Although scar size was variable (4.3 ± 6.6%LV mass), scar burden was at least moderate in 128 patients (24%), involving ≥5% of LV mass.

**Table 1:** Patient demographics

Characteristics	n = 4,110
Age (mean ± SD)	51.2 ± 18.7 years
Male gender	45.6%
Diabetes mellitus	10.4%
Hypertension	39.2%
Hyperlipidemia	29.0%
Smoking	21.8%
Family history of CAD	20.0%
History of heart failure	5.1%
LV ejection fraction (mean ± SD)	62.4 ± 7.5%

CAD = coronary artery disease,

LV = left ventricular ejection fraction

## Conclusions

The current investigation is the largest, multicenter CMR study of unrecognized scar. More than one in eight patients (13.2%) with normal contractile function and no history of MI undergoing routine CMR testing have unrecognized myocardial scar. Scar burden was often high with 24% having involvement of ≥5% of LV mass, despite preserved LVEF and no regional wall motion abnormalities. Given the poor outcomes associated with unrecognized scar, the data suggests that CMR is being underutilized for the identification of high-risk patients at U.S. hospitals.

